# Audiovisual biofeedback amplifies plantarflexor adaptation during walking among children with cerebral palsy

**DOI:** 10.1186/s12984-023-01279-5

**Published:** 2023-12-08

**Authors:** Alyssa M. Spomer, Benjamin C. Conner, Michael H. Schwartz, Zachary F. Lerner, Katherine M. Steele

**Affiliations:** 1 Department of Mechanical Engineering, University of Washington, Seattle, WA USA; 2 College of Medicine – Phoenix, University of Arizona, Phoenix, AZ USA; 3 Department of Orthopedic Surgery, University of Minnesota, Minneapolis, MN USA; 4 Department of Mechanical Engineering, Northern Arizona University, Flagstaff, AZ USA; 5 Gillette Children’s, 200 University Avenue East, Stop 490105, St. Paul, MN 55101 USA

**Keywords:** Biofeedback, Gait rehabilitation, Cerebral palsy, Motor adaptation

## Abstract

**Background:**

Biofeedback is a promising noninvasive strategy to enhance gait training among individuals with cerebral palsy (CP). Commonly, biofeedback systems are designed to guide movement correction using audio, visual, or sensorimotor (i.e., tactile or proprioceptive) cues, each of which has demonstrated measurable success in CP. However, it is currently unclear how the modality of biofeedback may influence user response which has significant implications if systems are to be consistently adopted into clinical care.

**Methods:**

In this study, we evaluated the extent to which adolescents with CP (7M/1F; 14 [12.5,15.5] years) adapted their gait patterns during treadmill walking (6 min/modality) with audiovisual (AV), sensorimotor (SM), and combined AV + SM biofeedback before and after four acclimation sessions (20 min/session) and at a two-week follow-up. Both biofeedback systems were designed to target plantarflexor activity on the more-affected limb, as these muscles are commonly impaired in CP and impact walking function. SM biofeedback was administered using a resistive ankle exoskeleton and AV biofeedback displayed soleus activity from electromyography recordings during gait. At every visit, we measured the time-course response to each biofeedback modality to understand how the rate and magnitude of gait adaptation differed between modalities and following acclimation.

**Results:**

Participants significantly increased soleus activity from baseline using AV + SM (42.8% [15.1, 59.6]), AV (28.5% [19.2, 58.5]), and SM (10.3% [3.2, 15.2]) biofeedback, but the rate of soleus adaptation was faster using AV + SM biofeedback than either modality alone. Further, SM-only biofeedback produced small initial increases in plantarflexor activity, but these responses were transient within and across sessions (p > 0.11). Following multi-session acclimation and at the two-week follow-up, responses to AV and AV + SM biofeedback were maintained.

**Conclusions:**

This study demonstrated that AV biofeedback was critical to increase plantarflexor engagement during walking, but that combining AV and SM modalities further amplified the rate of gait adaptation. Beyond improving our understanding of how individuals may differentially prioritize distinct forms of afferent information, outcomes from this study may inform the design and selection of biofeedback systems for use in clinical care.

**Supplementary Information:**

The online version contains supplementary material available at 10.1186/s12984-023-01279-5.

## Introduction

Mobility is critical for promoting independence and facilitating broad social, emotional, and cognitive development [1–3]. However, for individuals with cerebral palsy (CP), a nonprogressive neurologic injury in early development affects coordination and can lead to a variety of progressive secondary impairments, which may restrict walking capacity over time [4, 5]. To support mobility, individuals with CP commonly participate in treadmill-based gait training, which is designed to provide task-specific and high-intensity practice [6–9]. Treadmill training has demonstrated success in CP [7, 10, 11]; however, traditional protocols require high levels of therapist coaching for individuals to consistently recognize and correct movement error [7]. This not only increases the burden on therapists for longer training sessions, but may also attenuate response, as treatment goals may be vague or inconsistently reinforced [7].

Biofeedback is a promising extension of traditional treadmill training, as it enables *self-initiated* error correction during *goal-directed* practice [12]. Biofeedback systems are designed to provide the user with real-time information on a specific gait parameter (e.g., joint angle, force, movement accuracy, muscle activity) in relation to a desired performance to augment existing intrinsic (i.e., tactile or proprioceptive) pathways and enhance error recognition [12–14]. Commonly, systems are designed to present information visually, via displays or immersive environments, or aurally, using tones or music. Such biofeedback systems (collectively termed audiovisual (AV) biofeedback) have been used successfully to improve spatiotemporal parameters [15–18], joint power [19], joint kinematics [15], and muscle activity [20] in individuals with CP during walking. More recently, sensorimotor (SM) biofeedback systems, such as exoskeletons [21–24] or vibrotactile arrays [25], have been developed as a means of directly interfacing with intrinsic feedback pathways during walking to provide critical temporal and spatial information; this approach may be particularly valuable in CP where sensory processing is often impaired [4, 26]. To this end, Conner et al. recently demonstrated that providing SM biofeedback using a resistive ankle exoskeleton during walking elicited improvements in energy expenditure, walking speed, and motor control in a CP cohort [23, 27].

While the demonstrated success of these studies highlights the potential of using biofeedback in CP rehabilitation, combining modalities may further amplify outcomes [14]. Robust error recognition drives adaptation to perturbation, which suggests that presenting simultaneous extrinsic (e.g., AV feedback) and intrinsic (e.g., SM feedback) information using biofeedback may elicit greater adaptation and, therefore, increase the magnitude of responses [28–30]. This aligns with the theory of multisensory integration which states that providing information across disparate sensory path ways may induce a faster and more accurate response than unisensory stimuli and help prevent cognitive overload, particularly for complex tasks [14, 31–33]. Further, training with multisensory systems has been hypothesized to strengthen the connections between sensory areas and enhance future unisensory retrieval [14, 31]; practically, this means that individuals who train with multimodal biofeedback may be able to transition to simpler systems for longer-term reinforcement. These hypothesized benefits of multimodal over unimodal biofeedback during walking have been explicitly evaluated in nondisabled [34], stroke [35], and spinal cord injury populations [36]. However, in CP, there is still limited understanding of how the choice of biofeedback modality influences responses and, importantly, if there are advantages to presenting multimodal biofeedback during gait training [13, 21].

The aim of this study was to evaluate the extent to which individuals with CP adapt their gait patterns using AV and SM biofeedback when presented independently and in combination. Secondarily, we evaluated if response was retained or further enhanced immediately and two weeks after a multi-session acclimation protocol in which individuals received extended practice with both systems. We hypothesized that individuals with CP would be able to modify their gait patterns in response to each biofeedback modality, but that presenting AV and SM biofeedback in parallel would promote greater error recognition and, therefore, increase both the magnitude and rate of response compared to either modality alone. Secondarily, we hypothesized that following multi-session acclimation, participants would adapt gait more quickly and to a greater magnitude while using all modalities, suggesting they had retained knowledge of the biofeedback systems. As different biofeedback modalities may be more or less translatable to clinical environments due to cost or other constraints, results from this study will help guide the selection and integration of biofeedback into clinical gait training [13, 14, 37].

## Methods

### Participants

Eight individuals with CP were recruited to evaluate adaptation to multimodal biofeedback (Table 1). Prior to participation, informed consent was provided by participants and their caregivers, and the study protocol was reviewed and approved by the Northern Arizona University Institutional Review Board. Individuals were eligible to participate if they had: (1) the ability to walk for 10 min on a treadmill, using handrails as necessary, (2) the ability to follow verbal instructions, (3) no significant, uncorrected vision or hearing loss which would impact their ability to receive audio or visual cues, as determined by the research team, (4) no history of orthopedic surgery or lower-limb botulinum toxin injections within the last 6 months or had received clinician approval that any recent interventions had minimal effects on gait, and (5) no other conditions that would make participation unsafe, decided at the discretion of the research team.

**Table 1:** Participant demographics

	Gender	GMFCS level^a^	Diagnosis^b^	Age (yrs)	Height (m)	Mass (kg)	More-affected limb^c^	Walking speed (nd)^d^
P1	M	II	SD	18	1.76	62.14	R	0.31
P2	F	II	SD	12	1.45	42.41	L	0.28
P3	M	III	SD	13	1.58	42.18	R	0.22
P4	M	II	SD	15	1.65	60.78	L	0.25
P5	M	II	SD	13	1.50	39.01	R	0.31
P6	M	I	SH	12	1.43	39.46	R	0.32
P7	M	III	SD	15	1.56	68.95	L	0.23
P8	M	I	SH	16	1.65	65.77	R	0.27

^a^Gross Motor Function Classification System; Level determined by licensed physical therapist

^b^Diagnoses include: Spastic diplegia (SD), spastic hemiplegia (SH); Diagnosis reported by participant caregivers

^c^Reported by participants and confirmed by licensed physical therapist

^d^Nondimensional, calculated according to Hof A.L., 1996

### Experimental protocol

**Fig. 1:**
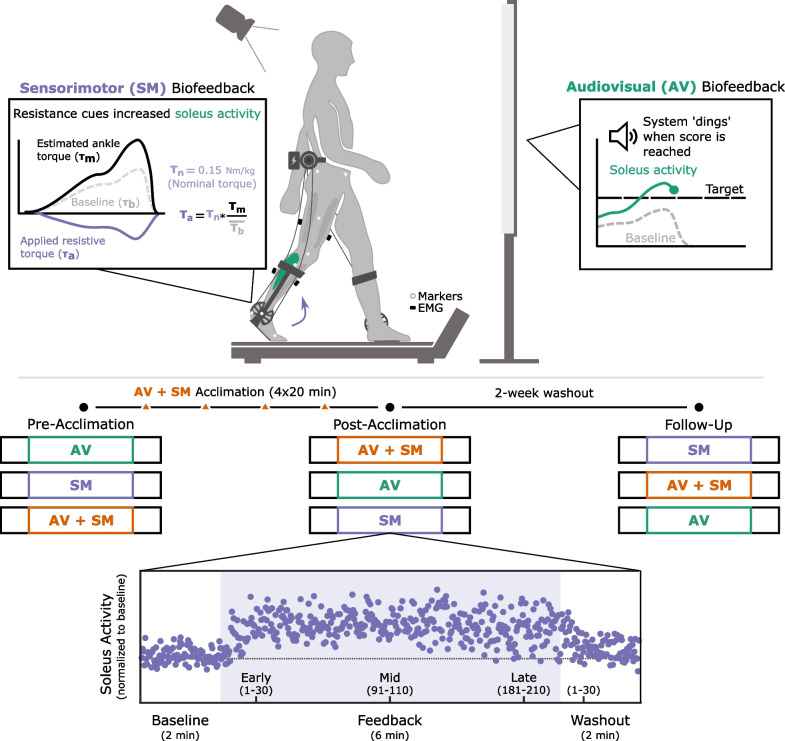
Experimental Protocol. Audiovisual (AV) biofeedback on soleus activity was provided for the more-affected limb alongside an auto-adjusting target score. Sensorimotor (SM) biofeedback was provided for the more-affected limb using an untethered ankle exoskeleton designed to impart a resistive ankle torque through stance, proportional to baseline values. Participants completed three data collection visits (pre-acclimation, post-acclimation, and follow-up), during which they walked with both biofeedback systems independently and in combination. Trials were pseudo-randomized within and between visits to ensure that feedback modalities were presented to each participant in a different order and control for fatigue and learning effects. Each trial was 10 min long and separated into baseline, feedback, and washout phases. All data analysis was performed for early (strides 1–30), mid (strides 91–110), and late (strides 181–210) feedback phases and washout (strides 1–30). Mean soleus activity for individual strides (purple dots) was normalized to baseline activity. Between the pre-acclimation and post-acclimation visits, participants completed four, 20-min acclimation sessions where they received additional practice with both systems

All participants walked on a treadmill at self-selected speed under three biofeedback conditions: (1) SM only, (2) AV only, and (3) combined AV + SM (Fig. 1). Both modalities were designed to directly target soleus activity on the more-affected limb, as reported by the participant (see sections "Sensorimotor biofeedback" and "Audiovisual biofeedback" for full system details). The plantarflexors were selected for this application, as they are critical for forward propulsion and commonly affected in CP [5, 38]. Further, providing biofeedback on muscle activity, rather than gait mechanics, is a marked deviation from most existing paradigms in CP, but may be more effective for eliciting changes higher up in the motor control hierarchy [13].

Each trial was structured to include baseline (1 min), feedback (6 min), and washout (1 min) phases and trials were separated by mandatory 5-min seated breaks. Across all phases, participants held onto the treadmill handrails for safety. Participants were given instructions before each trial began and cues at the transition between phases (e.g., between feedback and washout), but otherwise received no coaching in-trial to capture their natural response to each biofeedback modality. For each feedback phase, participants were instructed to focus on ‘pushing’ the treadmill belt behind them by activating their calf muscles and plantarflexing their ankle on their more affected side while maintaining an upright trunk. For both baseline and washout phases, the biofeedback systems were turned off and participants were instructed to walk in whatever way felt natural. The washout phase was included to capture any after-effects, which can provide insight into the extent of retention of the adapted gait pattern [39].

We evaluated participant response to each biofeedback modality across three visits: pre-acclimation, post-acclimation, and follow-up (Fig. 1). During the pre-acclimation visit, a licensed physical therapist confirmed the participant’s self-reported Gross Motor Function Classification System (GMFCS) level and more-affected limb, and evaluated knee (flexion/extension) and ankle (dorsi/plantarflexion) ranges-of-motion [40]. Between pre-acclimation and post-acclimation visits, participants performed four acclimation sessions where they received extended practice with both biofeedback systems. Each acclimation session consisted of two, 10-min treadmill bouts, during which participants walked with both systems turned on and received coaching and encouragement from the researchers on proper system engagement to maximize performance. All acclimation sessions were performed over a two-week period and separated by atleast one day to reduce participant fatigue. The follow-up visit was performed two weeks after the post-acclimation visit and used to evaluate whether individual responses to the biofeedback systems were maintained. Across all visits treadmill speed was held constant for each participant. Further, trials were pseudo-randomized within and across visits to ensure that each participant walked with the biofeedback modalities in a different order at each visit and thereby control for fatigue and learning effects.

### Sensorimotor biofeedback

Sensorimotor biofeedback (SM) was administered using a lightweight, battery-powered ankle exoskeleton, that has been previously evaluated in CP [22–24, 27, 41]. Briefly, this system uses DC motors, worn on a hip belt, to drive Bowden cables which actuate bilateral ankle assemblies (Fig. 1). The ankle assemblies consist of carbon fiber calf-cuffs and foot plates that can be sized to the individual to ensure optimal device fit. Force sensitive resistors, embedded in the footplates under the metatarsal heads, provide an on-board controller with a real-time estimate of the biological ankle torque and gait phase which are used to set the magnitude of the applied torque. During a baseline walking trial, the system is calibrated such that participants receive a nominal torque if their estimated biological ankle moment is similar in magnitude to average baseline values and receive proportionally higher or lower torque values otherwise; this design ensures that the device adapts to user input for every stride [22, 41, 42]. A custom Matlab interface communicates with the device via Bluetooth, enabling researchers to change the nominal torque value for both limbs and monitor system performance in-trial.

For the SM and AV + SM trials, the device was used to impart a *resistive* (i.e., dorsiflexion) moment at push-off on the more-affected limb to promote greater plantarflexor recruitment [22]. During the feedback phase across all three visits, the nominal resistive torque on the more-affected limb was set at 0.15 Nm/kg, normalized to participant mass, based on findings from pilot studies using the same device [22]. On the less-affected limb, the nominal torque was set to 0 Nm/kg (i.e., ‘zero-torque mode’) which effectively turned the ankle assembly into a hinge joint. During the baseline and washout phases, the same zero-torque mode was applied to the more-affected limb so as to capture the immediate transition between biofeedback and no-biofeedback walking without needing to stop the treadmill and doff the device. In the four acclimation sessions, held between the pre-acclimation and post-acclimation visits, torque was applied bilaterally, and the nominal torque value was increased from 0.1 Nm/kg (first session) to 0.2 Nm/kg (last session) in increments of 0.025 Nm/kg to maintain task challenge.

We elected to use a resistive exoskeletonto administer SM biofeedback because we believe that it has a unique advantage over assistive devices which are commonly used to the same ends [21, 41, 43, 44]. We hypothesized that the imposed resistive torque about the ankle would not only provide feedback on appropriate muscle activation timing, but would amplify existing movement error and thereby accelerate adaptation [29, 45, 46]. Further, the paradigm may also be particularly advantageous for translation into rehabilitation, as it encourages rather than supplants biological plantarflexor activity, promoting functional strength training and discouraging user ‘slacking’ [47].

### Audiovisual biofeedback

The audiovisual (AV) biofeedback system was custom designed in Matlab (Mathworks; Natick, MA). This system streams real-time electromyography (EMG) data from the soleus on the more-affected limb using Vicon Datastream (100 Hz; Denver, CO). These data are then smoothed using an 80 ms moving average filter and presented back to the user on a simple graphical display alongside a target score (Fig. 1). The system emits a ‘ding’ each time the target score is reached to notify the participant of a successful activation. To maintain task challenge and participant motivation, the target score is designed to be programmatically adjusted to keep user success rate between 50% and 75%, based on a sliding 10-stride window; if the participant’s success rate falls outside of these bounds (e.g., they reached the target score in only four of the last ten strides), the target score is either raised or lowered by 10%. This adaptive controller design not only ensures congruency with the adaptive SM system, but aligns with the challenge point framework, which hypothesizes that motor learning can be negatively impacted if task difficulty is too high, particularly for novice users [48].

### Data analysis

Across all three visits, surface EMG data were recorded bilaterally from the tibialis anterior, soleus, vastus lateralis, and semitendinosus, which were placed according to SENIAM guidelines (Noraxon; Scottsdale, AZ; 1000 Hz). EMG data were high-pass filtered (40 Hz; 4th order Butterworth), rectified, and low-pass filtered (10 Hz; 4th order Butterworth) to establish linear envelopes. A robust-PCA algorithm was then applied to the linear envelopes to remove any nonphysiological signal spikes due to sensor movement, and data was normalized to the 95th percentile of the first baseline phase evaluated [49, 50]. In parallel, lower-limb motion data were collected using a 10-camera motion capture system and the Vicon Lower-Limb Plug-in Gait marker set (Denver, CO; 100 Hz; Fig. 1). Three-dimensional joint kinematics were derived from marker data using the Vicon Plug-in Gait Dynamic pipeline [51, 52]. Secondarily, spatiotemporal parameters (e.g., stride length, stride width, step length) were calculated from marker data using the Gait Cycle Parameter Calculator in Vicon ProCalc. All EMG, kinematic, and spatiotemporal data were segmented into individual strides using the fore-aft heel marker and toe marker positions to determine heel-strike and toe-off, respectively.

Because we anticipated that participants may make bilateral modifications to the imposed unilateral perturbation, we characterized both intralimb and interlimb mechanics during each visit. Intralimb mechanics were defined as changes in (1) mean muscle activity, (2) co-contraction, (3) stride length, and (4) joint angles compared to baseline walking. Co-contraction was quantified for the soleus (SOL) and tibialis anterior (TA) using the co-contraction index (CCI) [53]:

1 $$CCI=\frac{2*{\int }_{1}^{t}Min\left(T{A}_{t}, SO{L}_{t}\right)}{{\int }_{1}^{t}T{A}_{t}+ {\int }_{1}^{t}SO{L}_{t}}$$

which measures the extent to which the integrated area of EMG data for both muscles overlaps across the gait cycle (t = 100 time points).

Interlimb mechanics were evaluated using step width and step length asymmetry. Step width was quantified as the medio-lateral difference between heel strikes on opposing limbs. Step length asymmetry was calculated as:

2 $${SLA}_{targeted}=\frac{{SL}_{targeted}- {SL}_{nontargeted}}{{SL}_{targeted}+ {SL}_{nontargeted}}$$

which captures step length differences between the limb with (SL_targeted_) and without (SL_nontargeted_) biofeedback [54], where step length is defined as the fore-aft difference between consecutive heel strikes on opposing limbs.

### Statistical analysis

To characterize transient responses to each biofeedback modality, we evaluated all interlimb and intralimb metrics at three instances within the feedback phase—early adaptation (strides 1–30), mid adaptation (strides 91–110), and late adaptation (strides 181–210)—as well as early washout (strides 1–30; Fig. 1). While some participants took more than 210 strides during the feedback phase, we elected to match stride numbers across all participants to control for the effect that repetition may have had on learning. For each participant, all interlimb and intralimb parameters were normalized to the first baseline phase evaluated for each visit.

We compared changes in interlimb and intralimb mechanics from baseline for each modality at each phase of biofeedback walking (i.e., early, mid, and late adaptation) using multiple Wilcoxon signed-rank tests. Secondarily, we compared if interlimb and intralimb mechanics differed significantly between biofeedback modalities for each phase using multiple Kruskal–Wallis tests; for those tests that reached significance, post-hoc Wilcoxon signed-ranks tests were used to perform pairwise comparisons. All comparisons to baseline walking and post-hoc tests were adjusted using a Holm-Šídák correction to account for multiple comparisons (n = 3). All statistical analyses were performed using the Matlab Statistical Toolbox with significance defined as p < α for α = 0.05 (MathWorks, Natick, USA). We report group-wise median values [IQR] unless otherwise indicated but have included individual responses to each feedback modality for select metrics in the Supplementary Materials.

## Results

### Pre-acclimation response

**Fig. 2:**
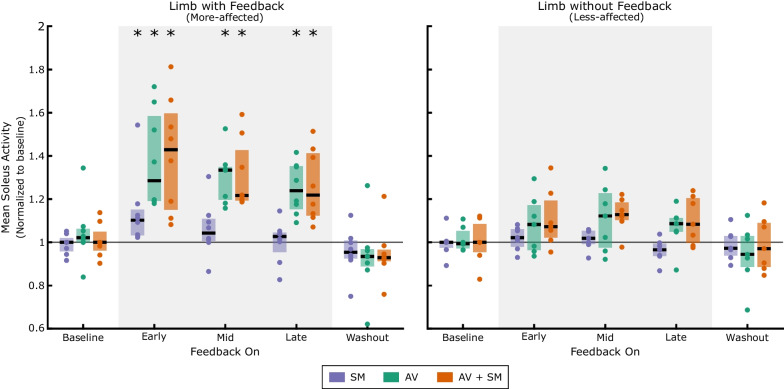
Mean soleus activity for both limbs during walking with sensorimotor (SM), audiovisual (AV), and combined (AV + SM) biofeedback at the pre-acclimation visit. Biofeedback was provided unilaterally on the more-affected limb. All data has been normalized to the first baseline phase attempted. For each participant, mean soleus activity during early (strides 1–30), mid (strides 91–110), and late (strides 181–210) adaptation and washout (strides 1–30) was calculated and is represented as individual dots. Box plots display the median (IQR) response. During both washout and baseline phases, the biofeedback systems were turned off. *indicates a statistical difference in soleus activity from baseline walking (Wilcoxon signed-rank tests with Holm-Šídák correction; α = 0.05)

When biofeedback was first turned on, participants were able to significantly increase mean soleus activity from baseline using each modality (Fig. 2, Additional file 1: Fig. S1; p = 0.02). However, the magnitude of changes in soleus activity with AV (median [IQR]: 28.5% [19.2,58.5]) and AV + SM (42.8% [15.1,59.6]) biofeedback were more than two times that of SM biofeedback alone (10.3% [3.2,15.2]). Further, while participants were able to maintain elevated soleus activity across the entire feedback phase using AV (late adaptation: 23.9% [15.4,35.2]) and AV + SM (late adaptation: 21.9% [12.4,41.3]) modalities, response to SM biofeedback was transient, returning to baseline by midsession (p > 0.20).

**Fig. 3:**
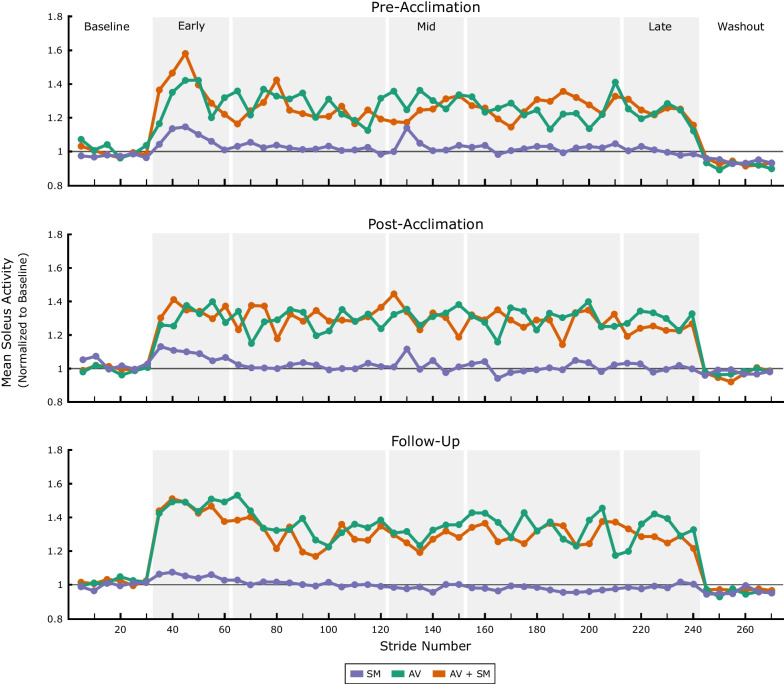
Time-course change in mean soleus activity for the more-affected limb during biofeedback walking. The median response to sensorimotor (SM) audiovisual (AV), and combined (AV + SM) biofeedback before (top) and after (middle) four acclimation sessions with AV + SM biofeedback and after a two-week follow-up (bottom) is displayed. All data has been normalized to the first baseline phase performed at each visit and averaged into 5-stride bins. During baseline and washout phases, the biofeedback systems were turned off

There was no statistical difference in the magnitude of mean soleus activity between AV and AV + SM biofeedback during the early, mid, and late adaptation phases of the pre-acclimation visit (p > 0.64), which suggests limited additive advantage of combining modalities. However, when examining early adaptation, individuals adapted more quickly to AV + SM biofeedback compared to the AV-only modality (Fig. 3). Within the first five strides of the biofeedback systems being turned on, participants increased soleus activity from baseline by 36.5% [8.2,78.1] using AV + SM (p = 0.046), whereas response to SM and AV biofeedback was not significantly different from baseline until individuals had taken atleast ten strides (p = 0.02).

Despite the observed in-session gains for each modality, soleus activity rapidly returned to baseline once the systems were turned off, indicating that there was limited short-term retention of the adapted gait patterns (p > 0.38 for all modalities during washout). In-session gains were also largely unilateral, as soleus activity was similar to baseline in the nontargeted limb (i.e., less-affected limb) across all modalities and phases of biofeedback walking (p > 0.09).

**Fig. 4:**
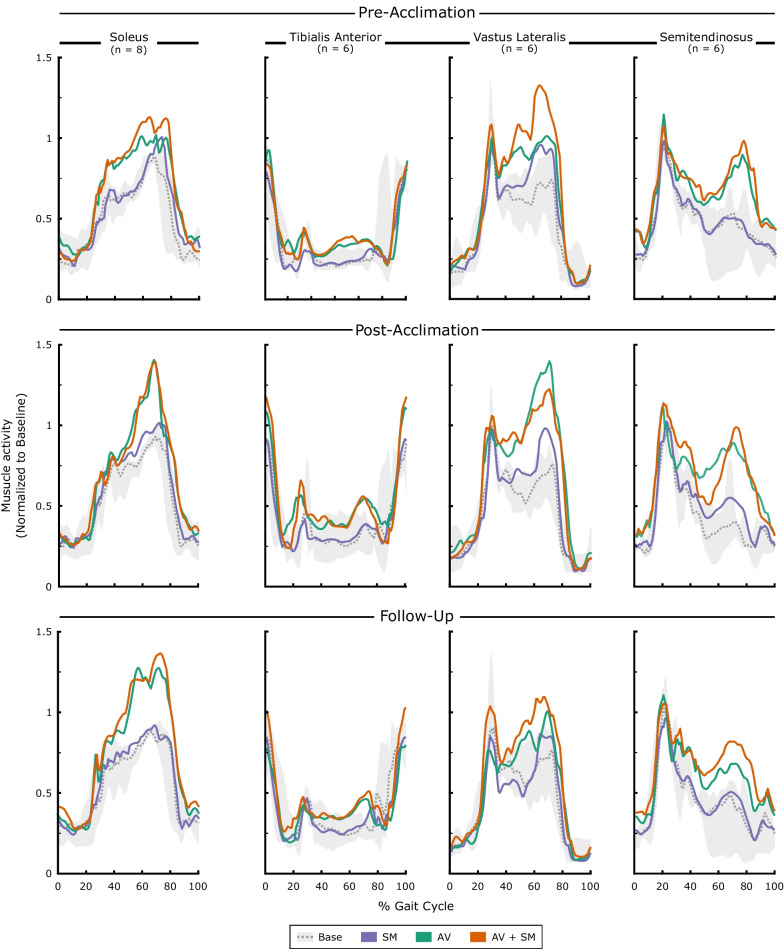
Changes in lower limb muscle activity during biofeedback walking. Median muscle activity for the more-affected limb during early adaptation (strides 1–30) to sensorimotor (SM), audiovisual (AV), and combined (AV + SM) biofeedback at the pre-acclimation (top), post-acclimation (middle), and follow-up (bottom) visits is displayed. For each visit, data were normalized to the 95th percentile of each participant’s first baseline phase. Baseline trends display median (IQR). Note that the trends shown for the tibialis anterior, vastus lateralis, and semitendinosus represent six of the eight participants tested, as two had to be removed due to loss of the vastus lateralis signal during collection

Although both modalities were designed to directly target soleus activity, participants altered their multi-muscle control strategy with biofeedback. The CCI between the tibialis anterior and soleus did not change significantly from baseline walking for any biofeedback modality (p > 0.68). However,  mean vastus lateralis activity (SM: 5.4% [1.8,42.2]; AV: 43.7% [14.3,61.1]; AV + SM: 70.5% [36.4,85.5]) and semitendinosus activity (SM: 4.2% [2.3,12.1]; AV: 46.5% [− 1, 65.3]; AV + SM: 57.6% [3.6, 100]) was elevated from baseline in the majority of participant for all modalities during early adaptation and remained elevated for AV and AV + SM modalities across the entire feedback phase, although none of these changes reached significance (Fig. 4; p > 0.09). Taken together, this suggests that individuals may have adopted proximal compensations to modulate soleus activity. Note that two participants were not included in this analysis, due to EMG signal losses during one session (vastus lateralis sensor for P3 at follow-up and P6 at pre-acclimation).

**Fig. 5:**
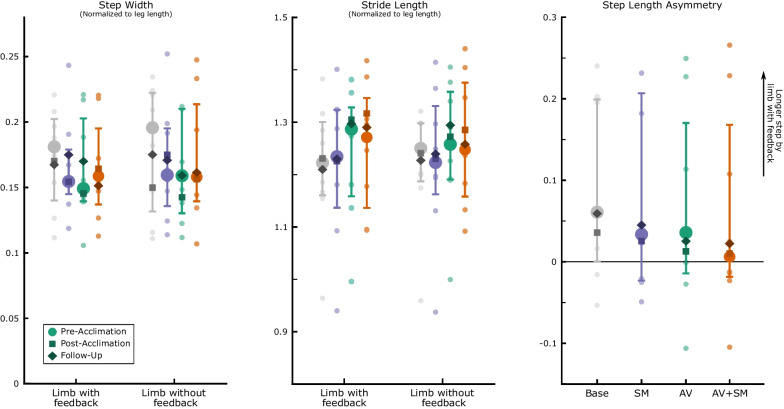
Spatiotemporal parameters during early adaptation (strides 1–30) to sensorimotor (SM), audiovisual (AV), and combined (AV + SM) biofeedback during the pre-acclimation visit. Plots portray median (IQR) as well as individual participant data (dots). A larger value for step length asymmetry (Eq. 2) indicates longer strides were taken on the limb targeted by biofeedback and a score near zero indicates symmetry. Step width and stride length have been normalized to leg length for each participant. Median post-acclimation (square) and follow-up (diamond) values are presented for all parameters. Across parameters, no significant differences were observed between each biofeedback modality and baseline or between modalities

While muscle activity was significantly modified during biofeedback walking, there were minimal changes in spatiotemporal parameters (Fig. 5). Though not significant, participants demonstrated small increases in stride length on the more-affected limb and small decreases in step width with biofeedback (p > 0.57 across all phases and modalities). There was also a small, but nonsignificant, decrease in step length asymmetry when walking with biofeedback compared to baseline (p > 0.22 across all phases and modalities). Individual changes in step length asymmetry during biofeedback walking are reported in Additional file 2: Fig. S2.

**Fig. 6:**
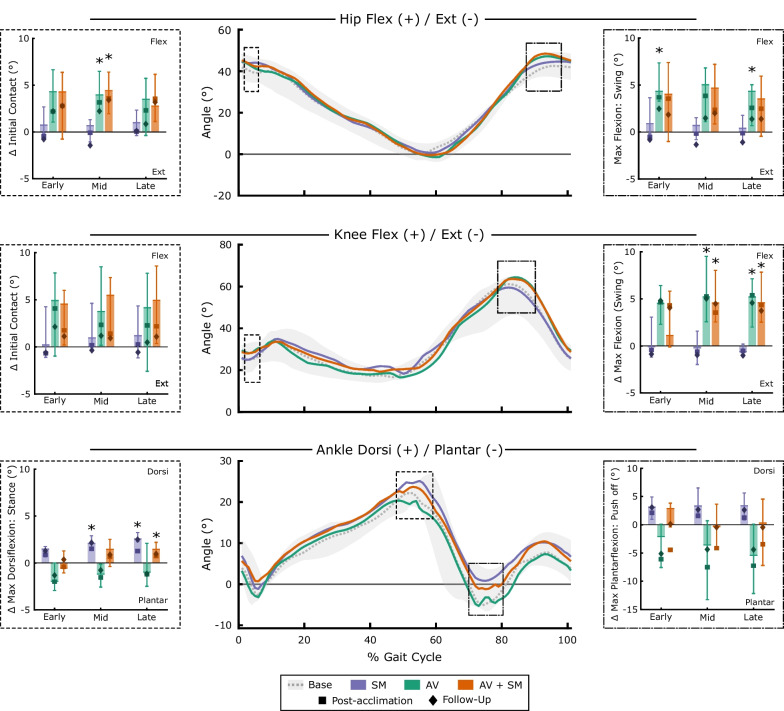
Sagittal plane kinematics for the hip, knee, and ankle on the more-affected limb during walking with sensorimotor (SM), audiovisual (AV) and combined (AV + SM) biofeedback. The middle panels show median trends for baseline and all biofeedback modalities during the late phase (strides 181–210) of the pre-acclimation visit. Baseline trends show median (IQR). Bar plots depict median (IQR) changes from baseline for key points within the gait cycle. Initial contact is defined as the mean value over the first 5% of the gait cycle. Median values for post-acclimation (square) and follow-up (diamonds) visits are also presented. Note that because there was interparticipant variability in the timing of maximum angles, there is some discrepancy between the bar plots and median kinematic trends. *denotes significant differences from zero, indicating a change from baseline values (α = 0.05; Wilcoxon signed-rank tests with Holm-Šídák correction)

In contrast to spatiotemporal parameters, participants altered kinematics in response to biofeedback (Fig. 6, Additional file 3: Fig. S3). Participants significantly increased hip flexion at initial contact during walking with AV and AV + SM modalities (p = 0.046; mid adaptation) compared to baseline. During swing, participants significantly increased maximum hip and knee flexion from baseline with AV biofeedback and maximum knee flexion with AV + SM biofeedback (p < 0.046). The largest discrepancy between AV + SM and AV modalities was seen at the ankle; SM and AV + SM biofeedback yielded significant increases in maximum ankle dorsiflexion in stance (p < 0.03), whereas there were small but nonsignificant decreases in dorsiflexion with AV biofeedback (p > 0.21). Plantarflexion during push-off was also greater with AV biofeedback than either SM or AV + SM modalities (Kruskal–Wallis; p < 0.04 for mid and late adaptation). No significant differences in pelvis or hip abduction kinematics were observed for any biofeedback modality (p > 0.11). Kinematic changes were also observed on the less-affected limb, despite the fact that muscle activity was not significantly altered from baseline. Individuals significantly increase knee flexion at initial contact for all biofeedback modalities (Additional file 4: Fig. S4; p = 0.046 for mid-adaptation). Further, AV and AV + SM biofeedback yielded increases in hip flexion in swing (p < 0.03 for early adaptation).

### Post-acclimation response

**Fig. 7:**
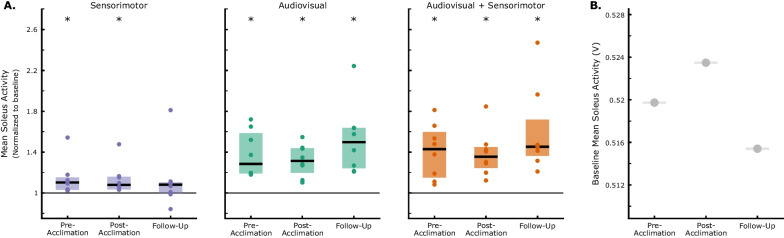
Changes in soleus activity following acclimation. **A** Mean soleus activity during early adaptation (strides 1–30) to sensorimotor (SM), audiovisual (AV), and combined (AV + SM) biofeedback at the pre-acclimation, post-acclimation, and follow-up visits. **B** For each visit, soleus activity during biofeedback walking was normalized to mean activity for the first baseline phase performed. * indicates a statistical difference in soleus activity from baseline (Wilcoxon signed-rank tests with Holm-Šídák correction; α = 0.05)

Following acclimation and at the two-week follow-up, participants demonstrated significant capacity to modulate soleus activity in response to biofeedback (Fig. 7). At the post-acclimation visit, mean soleus activity increased by 7.8% [3.5,15.8], 31.5% [19.8,43.8], 35.5% [24.4,45.0] relative to baseline in early adaptation using SM, AV, and AV + SM biofeedback, respectively (p = 0.02). However, similar to the pre-acclimation visit, response to SM biofeedback was not maintained at the post-acclimation visit (p > 0.11 for mid and late adaptation phases) and by the follow-up visit, participants demonstrated no response to the SM-only system across all phases (p > 0.25). Response to AV and AV + SM biofeedback for both visits was also almost immediate, as individuals demonstrated significant increases in soleus activity within the first five strides of walking with both modalities (p < 0.03 for post-acclimation and follow-up); this indicates that individuals maintained the ability to respond to both the AV and AV + SM biofeedback, even after two weeks without using either system. The timing of soleus activity also demonstrated changes following acclimation (Fig. 4). In particular, at post-acclimation, individuals predominantly modulated soleus activity at push-off using AV and AV + SM biofeedback whereas in the pre-acclimation visit, soleus activity increased across stance phase. However, despite these in-session changes in soleus recruitment, participants still quickly returned to baseline values during washout in both the post-acclimation (p > 0.68) and follow-up visits (p > 0.48). This suggests that, even following system acclimation, there was limited short-term retention of the adapted gait patterns.

There were notable differences in kinematic strategies across modalities following acclimation (Fig. 6). In contrast to the pre-acclimation visit, there were no significant differences in hip flexion at initial contact during the post-acclimation or follow-up visits (p > 0.11). Further, knee flexion in swing was only significantly elevated from baseline in early adaptation to AV biofeedback during post-acclimation and follow-up visits (p = 0.046) while hip flexion in swing was elevated for the same modality and phase during the follow-up visit only (p = 0.046). Participants maintained a similar strategy at the ankle using SM biofeedback before and after acclimation, as they significantly increased dorsiflexion through stance (p = 0.02 for all phases in post-acclimation and follow-up). However, in contrast to the pre-acclimation visit, response to the AV and AV + SM systems was similar in both the post-acclimation and follow-up visits (post-hoc pairwise comparison: p > 0.055), as individuals walked with greater plantarflexion at push-off using both systems. On the less-affected limb, participants increased knee flexion at initial contact for all modalities (p < 0.046 for mid adaptation; Additional file 4: Fig. S4) but did not demonstrate an increase in hip flexion during swing for any modality, in contrast to the pre-acclimation visit (p > 0.07). At the follow-up visit, participants also significantly increased plantarflexion at push-off on the less-affected limb across all modalities (p < 0.04 for early adaptation).

## Discussion

When presented with biofeedback, adolescents with CP were able to rapidly modify soleus activity during walking; however, response varied considerably depending on the modality used. Both AV and AV + SM biofeedback elicited similar changes in soleus activity within a single session, but individuals adapted more quickly to the combined modality. This finding partially supports our original hypothesis, as combining modalities advantageously affected the rate but not magnitude of response. Response to both systems was also generally consistent immediately following multi-session acclimation and at a two-week follow-up. In contrast, response to SM biofeedback alone unexpectedly decreased within sessions and across subsequent exposures, to the point that it wholly disappeared by the follow-up visit. Regardless of these differences between modalities, response was entirely contingent on the presence of biofeedback in all cases, as individuals showed minimal retention of the adapted patterns once biofeedback was turned off.

That individuals were able to modulate soleus activity in response to biofeedback indicates that these paradigms may be clinically valuable, even though in-session gains were not retained. Plantarflexor weakness is a common downstream effect of neurologic injury and has been associated with gross motor function and gait pathology in CP [55, 56]. As altered gait patterns impact bony alignment and increase joint pain, which may lead to the eventual degradation of independent walking, improving muscle strength has been a key target in CP rehabilitation [10, 57–59]. While traditional weightlifting programs are commonly used to this end, outcomes remain limited as these strategies do not promote strength building within the context of gait [10, 60]. Therefore, having an adaptive paradigm that can promote targeted and individualized plantarflexor strength training during walking may be a valuable task-specific addition to rehabilitation.

The noted discrepancy between response to the SM-only system and the other modalities could stem from multiple sources and provides insight into the mechanisms by which individuals with CP adapt to biofeedback. As both systems presented distinct afferent information, individuals could have been differentially prioritizing AV and SM cues. This aligns with the specificity-of-learning hypothesis which suggests that the sources of error information that are deemed most reliable drive learning whereas all other potential information sources are ignored [14, 61]. Because intrinsic feedback pathways (i.e., proprioception, sensation) are commonly affected in CP, it is likely that individuals may automatically weight other sensory information, such as vision, more highly during error correction and perturbation recovery tasks. Prior studies in older adults and individuals with multiple sclerosis lend credence to this hypothesis, as both groups have been shown to be more susceptible to visual perturbation during walking which the authors postulate is likely due to somatosensory deficits [62, 63]. Sexton et al. made a similar conclusion in nondisabled individuals during reaching tasks, in which they showed that vision may be prioritized over proprioception when the latter is made artificially unreliable [64].

Responses also likely reflected fundamental differences in biofeedback system design. Because the sensorimotor biofeedback system resisted the desired motion of the ankle, it may have been inherently less intuitive for users to engage with than the audiovisual system which responded in the same direction as the task instructions (i.e., greater muscle activity caused the line on the screen to go up) Further, as the sensorimotor system actively made walking more challenging, individuals were likely more prone to compensate kinematically to bypass the effects of the device. This can be observed in the ankle kinematics, in which participants demonstrated greater dorsiflexion through stance with SM biofeedback compared to the other modalities and baseline walking; similar observations have been reported by Conner et al. in response to the SM system used in this study [65]. While participants were instructed to resist the device throughout the biofeedback walking phase, without the continual prompting afforded by the AV system, they likely prioritized gait strategies which reduced overall effort. This highlights an inherent limitation of resistive SM biofeedback paradigms in isolation and points to the benefit of using parallel AV prompting to continuously focus attention and reinforce desired device engagement.

While the overall magnitude of response to the AV and AV + SM systems were similar, we did note small but significant differences in the rate at which individuals responded to both systems. In line with our original hypothesis, individuals walking with AV + SM biofeedback modified their soleus activity almost immediately whereas adaptation occurred more slowly when the systems were used independently. This suggests that AV + SM biofeedback may have enhanced error recognition and subsequently prompted more rapid movement correction. Prior studies comparing audio and visual biofeedback during gait have reported similar findings, as they have demonstrated that nondisabled individuals and stroke survivors alter gait to a greater extent when information is applied multimodally [34, 35]. Further, Yen et al. demonstrated that a combination of visual biofeedback and resistance, designed to augment proprioceptive information, elicited greater and longer lasting changes in stride length in individuals with incomplete spinal cord injury than either system independently [36]. Counter to these studies, the apparent advantage of the AV + SM biofeedback system used in this study was not maintained past the beginning of the trial; however, this is likely the result of the rapid attenuation of response to the SM modality, as previously discussed.

Individuals also employed distinct kinematic strategies to elicit changes in soleus activity with AV and AV + SM biofeedback. In particular, during the pre-acclimation visit we noted that individuals walking with the AV-only system exhibited greater plantarflexion through push-off than either of the other modalities. Although improving range of motion is generally a target in CP rehabilitation [5], this may have contributed to the proximal compensatory strategies observed during AV walking; increased plantarflexion in early swing may have required individuals to increase hip and knee flexion to create sufficient toe clearance. This highlights a shortcoming of the AV biofeedback system, as it may have provided insufficient information to communicate both the desired magnitude and timing of gait changes [14]. In contrast, the SM-only system applied resistance during the stance phase, which may have provided more specific cues on appropriate plantarflexion timing, decreasing the need for compensation at the hip through swing.

Acclimation to both biofeedback systems also affected how individuals responded to each modality. At the post-acclimation visit, participants increased soleus activity almost immediately after the systems were turned on and the changes were predominantly isolated to push off which suggests that they had learned how to effectively engage with both systems. This observation aligns with previous studies on motor adaptation which have demonstrated that individuals respond more quickly following repeated exposures to a perturbation, indicating that the central nervous system has stored knowledge of the novel environment [39, 66]. Participants also appeared to become more adept at overcoming the resistive torque of the SM system following acclimation, as ankle kinematics during push-off became more similar during walking with the AV and AV + SM. Interestingly, these noted changes at the ankle were not accompanied by consistent changes in hip and knee kinematics, as both were largely unchanged from baseline following acclimation. This implies that participants had learned to modulate soleus activity without simultaneously adopting full-limb compensatory strategies as was observed during the pre-acclimation visit.

Despite the in-session improvements observed following acclimation, individuals did not demonstrate retention of the adapted patterns once biofeedback was turned off. Given the length of each walking bout and the number of sessions evaluated in this study, we did not anticipate that there would be significant transfer of in-session gains. However, because response to AV and AV + SM biofeedback did not notably decrease, even after a two-week washout, there may be larger carry-over effects if an extended training program is used. Prior work by Conner et al. demonstrated that individuals with CP had measurable improvements in soleus recruitment, co-contraction, and energy expenditure during overground walking following a 10–12 session training program using SM biofeedback independently [23, 27]. Because we saw that AV biofeedback amplified response, we anticipate that training outcomes could be even greater with AV + SM training. Transfer may have also been affected by the manner in which biofeedback was administered within each visit. Prior evidence has suggested that both the timing and the frequency with which cues are presented may influence retention of adapted gait patterns [14]. We elected to provide all biofeedback concurrently during walking, as evidence suggests that continual reinforcement of the desired trajectory is critical in the early stages of learning a complex motor task [14]. However, participants likely developed dependence on the biofeedback system to prompt gait changes. Employing fading or intermittent biofeedback paradigms, in which progressively less guidance is provided with skill acquisition, may force participants to rely more heavily on their own error estimation and correction pathways which, in turn, may promote longer-term transfer [14, 67–69]. Understanding the factors that influence if and how gait adaptations are retained outside of the context of biofeedback training will be a critical area for future research if it is to be considered a viable rehabilitation strategy for individuals with CP.

Finally, across visits and biofeedback modalities, we noted a high level of interparticipant variability in response (Additional file 1: Fig. S1, Additional file 2; Fig S2, Additional file 3: Fig. S3). This suggests that beyond the choice of modality, there are many other participant and system-level factors that may affect how an individual interacts with biofeedback. Because we were using a multimodal paradigm, which included a resistive ankle exoskeleton, individuals’ selective motor control about the ankle, muscle strength and fatiguability, and proprioception may have all influenced outcomes. Prior work has also indicated that an individual’s capacity to modify feedforward gait strategies in response to perturbation and temporarily store the adapted pattern is contingent on both the severity and location of the primary neurologic injury [70]. While we did not note a significant correlation between functional ability (GMFCS level) and the magnitude of response to the biofeedback paradigms (Pearson’s correlation; p > 0.09 for all modalities) in our small sample, prior work by our team has demonstrated that GMFCS level is one of many factors which has a direct effect on response to the SM system used in this study, warranting additional investigation [65]. Other participant-level factors such as an individual’s dual-task capacity or age may have further influenced their ability or motivation to effectively engage with the system and modify their gait pattern accordingly. Response following the acclimation visit may have also differed if acclimation protocols had been individualized; for this application all individuals underwent the same four acclimation sessions with AV + SM biofeedback which was likely insufficiently challenging for some individuals and overtaxing for others [48]. Given this complexity, there is a need to develop robust analytical techniques to comprehensively model these participant-device interactions [65]. To this end, prior studies have explored using causal modeling frameworks to identify factors which directly affect gait mechanics following surgical intervention and elevated energy costs in CP [71–73]. Applying a similar strategy to understand biofeedback outcomes may inform how systems can be designed to optimize responses and aid in selecting candidates for training.

To our knowledge, this is the first study to compare how the choice of modality may influence response to biofeedback among individuals with CP; however, there are limitations to our approach that need to be considered. We evaluated a small and highly heterogeneous sample of individuals. While this demonstrated that biofeedback may be beneficial for a broad user base, it did limit our statistical power. We also did not compare response to the audio or visual systems independently. This was motivated by the fact that we were primarily interested in contrasting the effects of intrinsic versus extrinsic biofeedback systems rather than provide a comprehensive understanding of response to many distinct forms of extrinsic feedback. But, as audio biofeedback may be particularly advantageous for administering training during overground walking, characterizing how response may change if visual information is also omitted is an important area for future research. Further, we fixed speed across sessions to control for any potential confounding effects that changes in speed may have on kinematics and muscle activity. However, this may have inadvertently introduced a ceiling effect on the extent to which individuals could effectively modulate soleus activity. We were also unable to directly compare the magnitude of response across the three visits due to the manner in which we normalized EMG data. We elected to normalize all activity to the first baseline phase tested within each visit, rather than a maximum voluntary isometric contraction (MVIC), given the challenges of collecting reliable MVICs in children with neuromuscular disorders [74]. As such, there were differences in the baseline activity used for normalization which limited intersession comparison (Fig. 7). The location of the calf cuff on the ankle exoskeleton also made it challenging and uncomfortable to measure gastrocnemius activity. Given the biarticular nature of the gastrocnemius, monitoring its activity during biofeedback walking would provide salient information on how individuals were compensating in response to each paradigm and is, therefore, a critical addition to future work. Understanding the extent that individuals can increase gastrocnemius activity in response to these feedback paradigms also has significant clinical implications, as the gastrocnemius is almost universally affected in CP and a target for most existing interventions [5]. Finally, as previously described, this study was not designed to specifically evaluate training outcomes due to time and resource constraints. As the goal of biofeedback training in CP is to ultimately improve everyday function, evaluating each modality's effect on gait outcomes following a longer training protocol is a critical next step of this work.

## Conclusion

Evaluating multi-session response to AV and SM biofeedback systems demonstrated how individuals with CP differentially prioritize distinct intrinsic and extrinsic cues. This analysis revealed that individuals are capable of modulating muscle activity in response to biofeedback, but both the rate and magnitude of adaptation is sensitive to the modality used. Specifically, we found that AV biofeedback consistently amplified participant responses whereas responses to SM biofeedback were more transient. Secondarily, we observed that individuals became more adept at responding to biofeedback with repeated exposure. Evaluating how the choice of biofeedback modality affects response is a necessary first step in informing future system design such that biofeedback-augmented gait training can become an efficacious clinical strategy to improve mobility in CP.

### Supplementary Information

**Additional file 1: Figure S1.** Mean soleus activity for the more-affected limb during walking with sensorimotor (SM), audiovisual (AV), and combined (AV + SM) biofeedback for each participant (P1-P8). Data is displayed for the pre-acclimation visit only and has been normalized to the first baseline walking phase attempted. For each participant, mean soleus activity during early (strides 1–30), mid (strides 91–110), and late (strides 181–210) adaptation and washout (strides 1–30) phases is represented as individual dots.

**Additional file 2: Figure S2. **Mean step length asymmetry during walking with sensorimotor (SM), audiovisual (AV), and combined (AV + SM) biofeedback for each participant (P1-P8). A larger value indicates longer strides were taken on the limb targeted by biofeedback and a score near zero indicates symmetry (Eq. 2). Data represents baseline, early (strides 1–30), mid (strides 91–110), and late (strides 181–210) adaptation, and washout (strides 1–30) phases during the pre-acclimation visit.

**Additional file 3: Figure S3.** Mean sagittal plane kinematics for the hip, knee, and ankle on the more-affected limb during baseline and biofeedback walking at the pre-acclimation visit for each participant (P1-P8). Data represents the late adaptation phase (strides 181–210) for sensorimotor (SM), audiovisual (AV), and combined (AV + SM) biofeedback modalities as well as baseline walking. Baseline trends show mean (95% confidence interval).

**Additional file 4: Figure S4.** Sagittal plane kinematics for the hip, knee, and ankle on the less-affected limb during walking with sensorimotor (SM), audiovisual (AV), and combined (AV + SM) biofeedback. Middle panels show median trends for baseline and all biofeedback modalities during the late adaptation phase (strides 181–210) of the pre-acclimation visit. Baseline trends show median (IQR). Bar plots depict median (IQR) changes from baseline for key points within the gait cycle. Initial contact is defined as the mean value over the first 5% of the gait cycle. Median values for post-training (square) and follow-up (diamonds) visits are also presented on the bar plots. Note that because there was interparticipant variability in the timing of maximum angles, there is some discrepancy between the bar plots and median kinematic trends. *denotes significant differences from zero, indicating a change from baseline values (α = 0.05; Wilcoxon signed-rank tests with Holm-Šídák correction).

## Data Availability

The datasets used for the current study are available from the corresponding author on reasonable request.

## Abbreviations

CP: Cerebral palsy
AV: Audiovisual
SM: Sensorimotor
SD: Spastic diplegia
SH: Spastic hemiplegia
GMFCS: Gross Motor Function Classification Scale
TA: Tibialis anterior
GAS: Medial gastrocnemius
VL: Vastus lateralis
ST: Semitendinosus
Robust-pCA: Robust principal component analysis
CCI: Co-contraction index
SLA: Step length asymmetry
IQR: Interquartile range
MVIC: Maximum voluntary isometric contraction
