# Supplementary figures and images for: Audiovisual biofeedback amplifies plantarflexor adaptation during walking among children with cerebral palsy
Source: J Neuroeng Rehabil. 2023 Dec 8;20:164. doi: 10.1186/s12984-023-01279-5 (PMC10704679; doi:10.1186/s12984-023-01279-5)

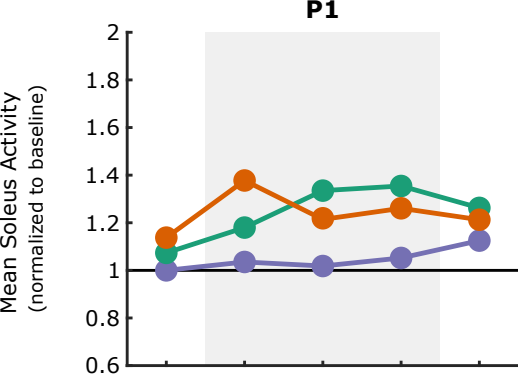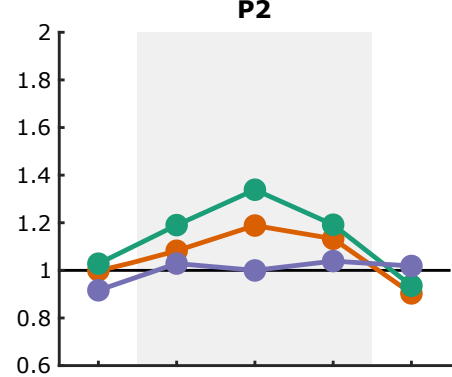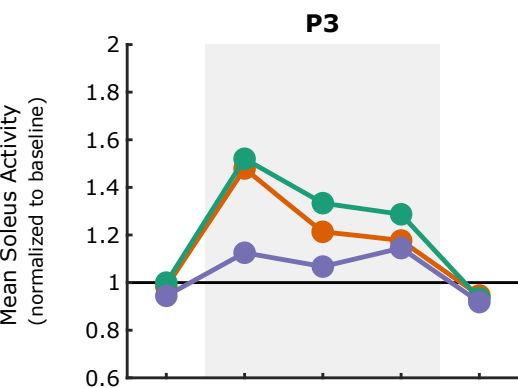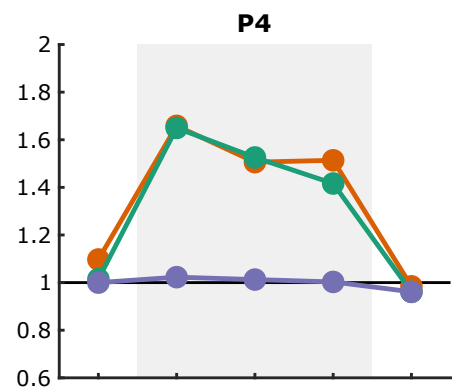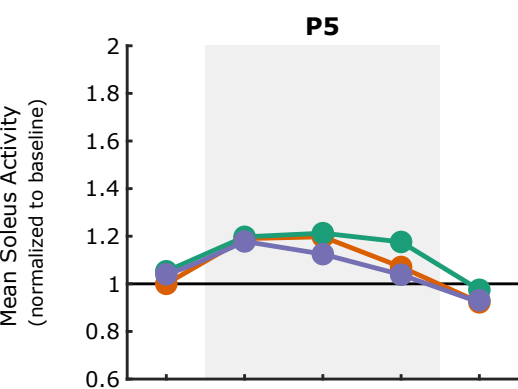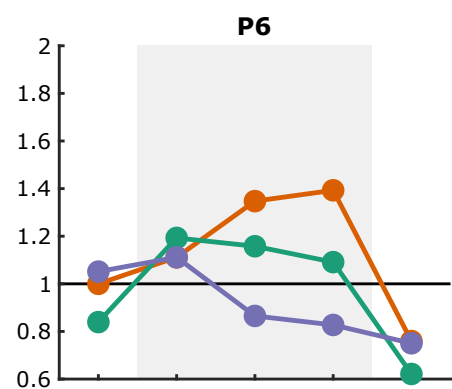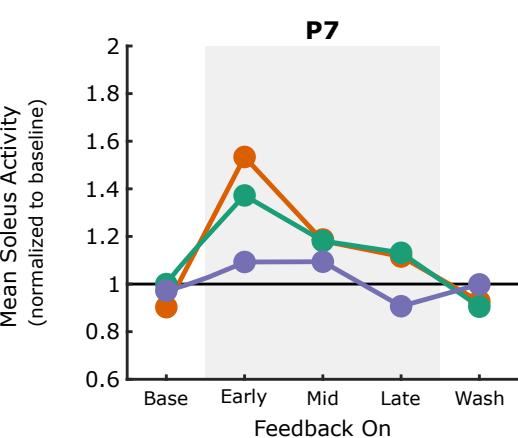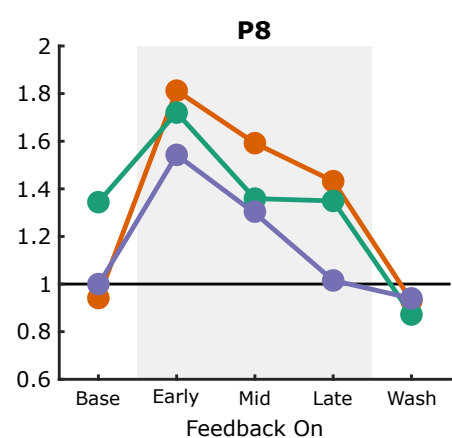

SM AV AV + SM

Supplement: Supplementary file 1 — Additional file 1: Figure S1. Mean soleus activity for the more-affected limb during walking with sensorimotor (SM), audiovisual (AV), and combined (AV + SM) biofeedback for each participant (P1-P8). Data is displayed for the pre-acclimation visit only and has been normalized to the first baseline walking phase attempted. For each participant, mean soleus activity during early (strides 1–30), mid (strides 91–110), and late (strides 181–210) adaptation and washout (strides 1–30) phases is represented as individual dots. [file 12984_2023_1279_MOESM1_ESM.pdf]

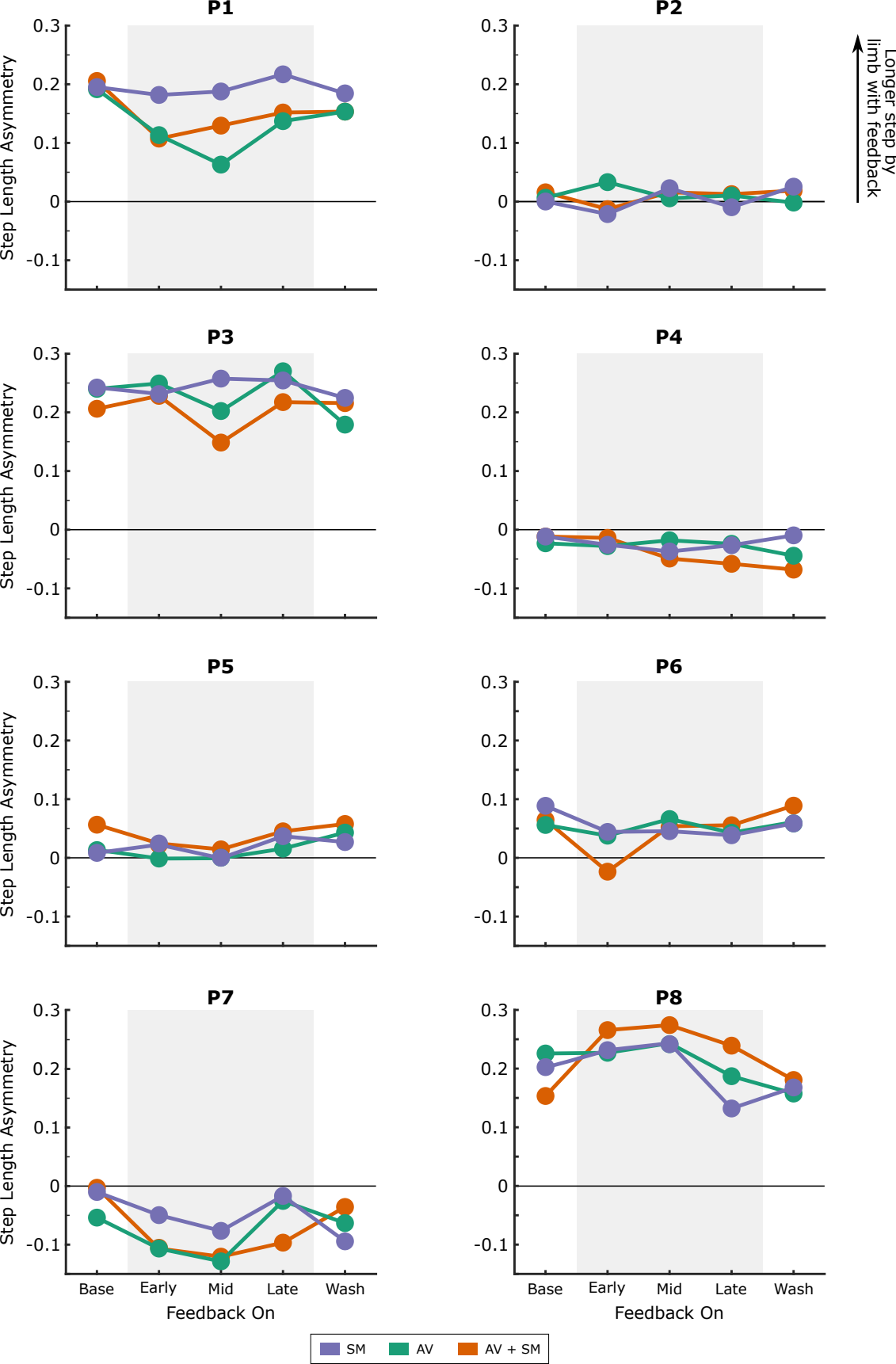

Supplement: Supplementary file 2 — Additional file 2: Figure S2. Mean step length asymmetry during walking with sensorimotor (SM), audiovisual (AV), and combined (AV + SM) biofeedback for each participant (P1-P8). A larger value indicates longer strides were taken on the limb targeted by biofeedback and a score near zero indicates symmetry (Eq. 2). Data represents baseline, early (strides 1–30), mid (strides 91–110), and late (strides 181–210) adaptation, and washout (strides 1–30) phases during the pre-acclimation visit. [file 12984_2023_1279_MOESM2_ESM.pdf]

# Late Adaptation (Strides 180-210)

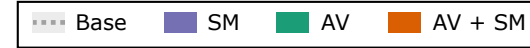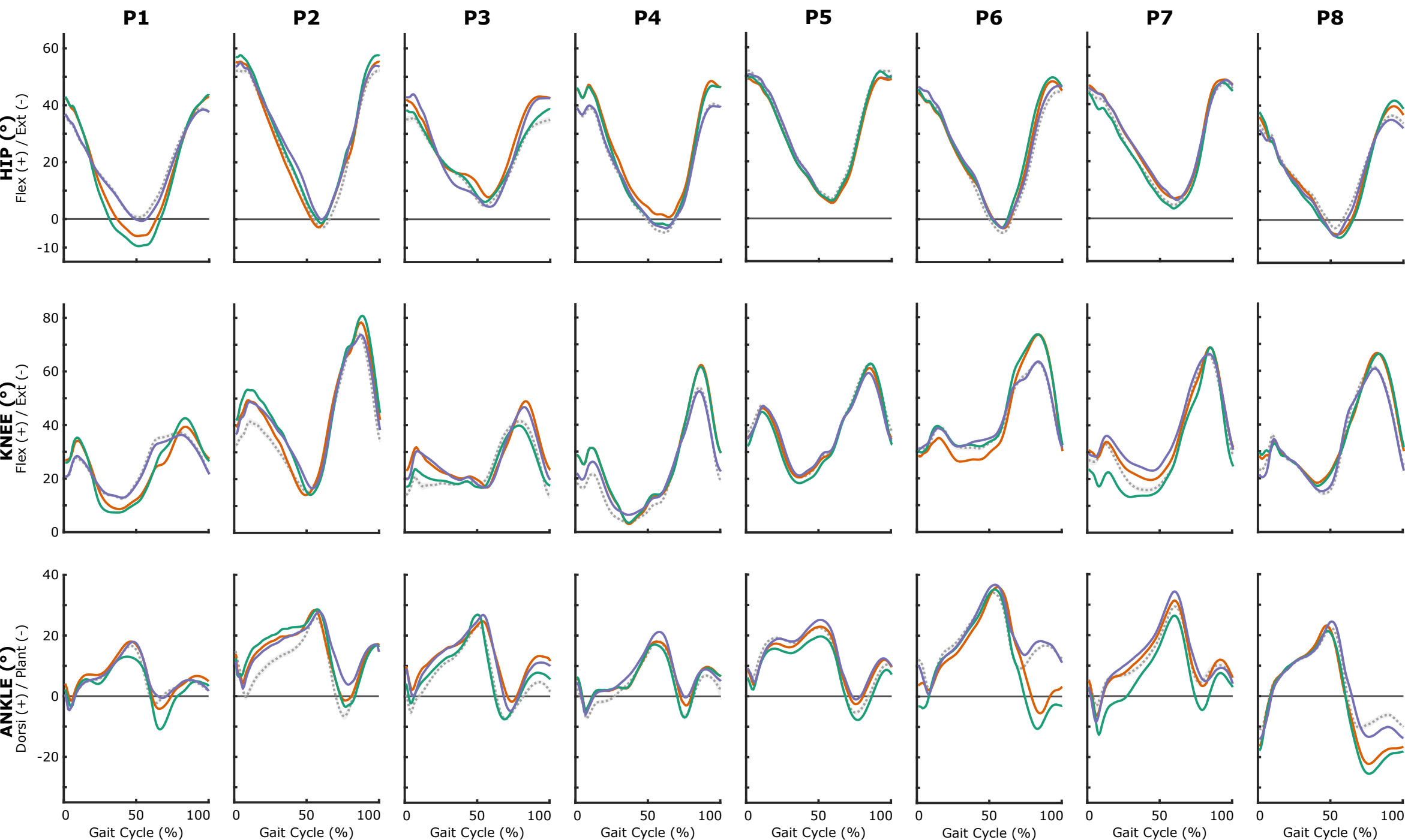

Supplement: Supplementary file 3 — Additional file 3: Figure S3. Mean sagittal plane kinematics for the hip, knee, and ankle on the more-affected limb during baseline and biofeedback walking at the pre-acclimation visit for each participant (P1-P8). Data represents the late adaptation phase (strides 181–210) for sensorimotor (SM), audiovisual (AV), and combined (AV + SM) biofeedback modalities as well as baseline walking. Baseline trends show mean (95% confidence interval). [file 12984_2023_1279_MOESM3_ESM.pdf]

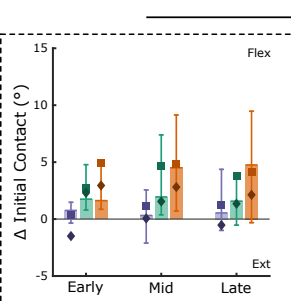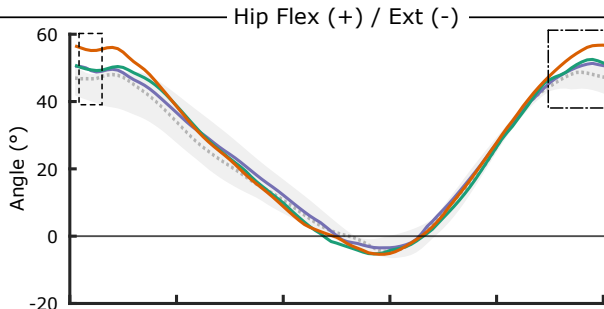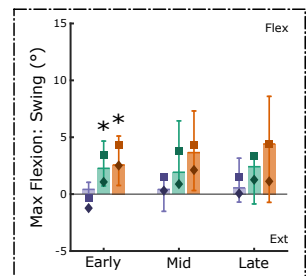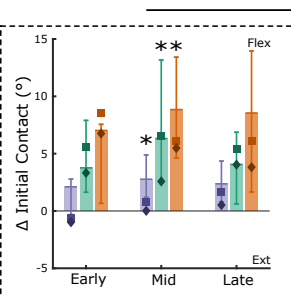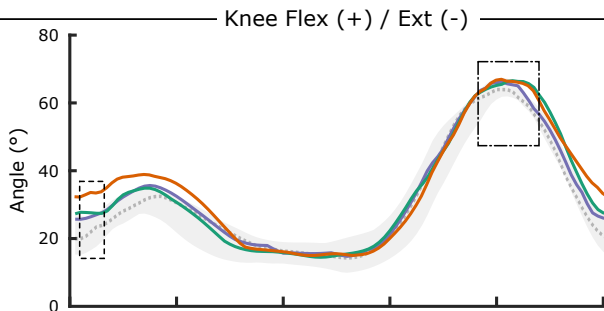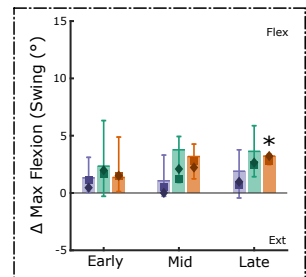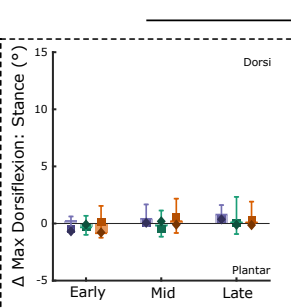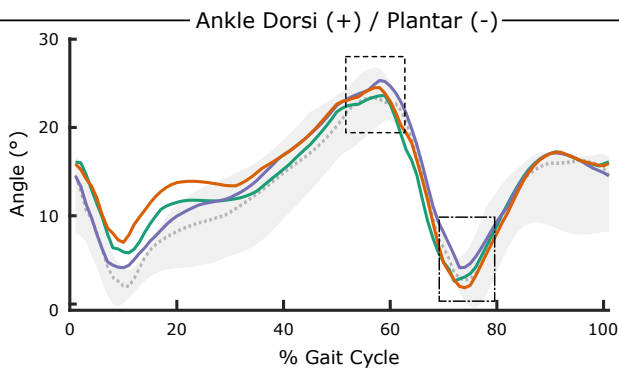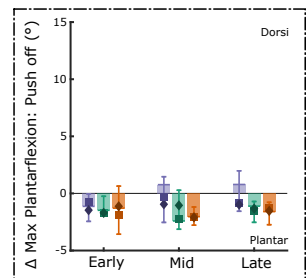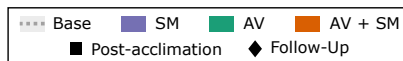

Supplement: Supplementary file 4 — Additional file 4: Figure S4. Sagittal plane kinematics for the hip, knee, and ankle on the less-affected limb during walking with sensorimotor (SM), audiovisual (AV), and combined (AV + SM) biofeedback. Middle panels show median trends for baseline and all biofeedback modalities during the late adaptation phase (strides 181–210) of the pre-acclimation visit. Baseline trends show median (IQR). Bar plots depict median (IQR) changes from baseline for key points within the gait cycle. Initial contact is defined as the mean value over the first 5% of the gait cycle. Median values for post-training (square) and follow-up (diamonds) visits are also presented on the bar plots. Note that because there was interparticipant variability in the timing of maximum angles, there is some discrepancy between the bar plots and median kinematic trends. *denotes significant differences from zero, indicating a change from baseline values (α = 0.05; Wilcoxon signed-rank tests with Holm-Šídák correction). [file 12984_2023_1279_MOESM4_ESM.pdf]
